# The Multisite SuperAging Research Initiative: Enrollment and Scientific Progress

**DOI:** 10.1002/alz.090906

**Published:** 2025-01-03

**Authors:** Emily J Rogalski, Adam Martersteck, Angela C Roberts, Matthew J. Huentelman, Ozioma C Okonkwo, Phyllis Timpo, Hannah Peirce, Ethan Flowers, Sophia Moore, Chandler Zolliecoffer, Rhiana Schafer, Rebecca Devine, Janessa Engelmeyer, Changiz Geula, Elizabeth Addison, Ignazio Piras, Antoine R Trammell, Gabrielle A Lincoln, Felicia Goldstein, Karen Van Ooteghem, Bill McIlroy, Robert Bartha, Elizabeth Finger, Ivan Culum, Andrew Lim, Richard H Swartz, Nathan Pruneau Gill, Amanda Cook Maher

**Affiliations:** ^1^ Healthy Aging & Alzheimer’s Research Care (HAARC) Center, Healthy Aging & Alzheimer’s Research Care (HAARC) Center, The University of Chicago, Chicago, IL USA; ^2^ University of Chicago, Chicago, IL USA; ^3^ Western University, London, ON Canada; ^4^ Neurogenomics Division, Translational Genomics Research Institute, Phoenix, AZ USA; ^5^ Center for Health Disparities Research, Department of Medicine, School of Medicine and Public Health (SMPH), University of Wisconsin‐Madison, Madison, WI USA; ^6^ Northwestern University Feinberg School of Medicine, Chicago, IL USA; ^7^ The Translational Genomics Research Institute (TGen‐ an Affiliate of City of Hope), Phoenix, AZ USA; ^8^ Emory University Goizueta Alzheimer’s Disease Research Center, Atlanta, GA USA; ^9^ University of Michigan, Ann Arbor, MI USA; ^10^ Emory University School of Medicine, Atlanta, GA USA; ^11^ University of Waterloo, Waterloo, ON Canada; ^12^ Department of Clinical Neurological Sciences, University of Western Ontario, London, ON Canada; ^13^ Sunnybrook Health Sciences Centre, Toronto, ON Canada; ^14^ Research Program on Cognition & Neuromodulation Based Interventions, Ann Arbor, MI USA

## Abstract

**Background:**

The multisite SuperAging Research Initiative (SRI) was established in 2021 to identify resilience and resistance factors promoting cognitive healthspan through a harmonized multidisciplinary protocol with prospective data collection. The designation of SuperAger is reserved for individuals age 80+ with episodic memory performance that is at least average for those 2‐3 decades younger. Research studies of this relatively uncommon phenotype allow for investigations of fundamental importance to the neurobiology of brain aging, resilience, resistance, and avoidance of cognitive decline related to “average aging” and more severe impairments associated with Alzheimer’s and related dementias (ADRD). The SRI is focused on increasing participant diversity and deep phenotyping through the enrollment of 500+ participants into a multi‐component longitudinal protocol. This presentation will summarize the engagement, recruitment, and baseline characteristics as well as the initial scientific findings from the cohort.

**Method:**

Enrollment and harmonized data collection occurs across North American sites in the U.S. and Canada. The SRI includes three Cores (Administrative/Biostatistics, Clinical/Imaging, and Biospecimen/Neuropathology) and two Research Projects. The Core infrastructure provides behavioral, health, genetic, environmental, socioeconomic, psychosocial, neuropsychologic, neuroimaging, and neuropathologic measurements. Project 1 uses state‐of‐the‐art wearable technology to obtain quantitative measurements of daily life (e.g., sleep, physical activity, and social engagement) to determine whether SuperAgers have relatively preserved physiologic and behavioral ‘complexity’. Project 2 uses transcriptomic, genetic, and protein profiling to examine central and peripheral immune and inflammatory system parameters.

**Result:**

Community‐engaged research practices have been implemented, yielding enrollment of >170 participants (ages 80‐108) into the multicomponent harmonized protocol. Approximately half of the participants have been co‐enrolled into Project 1 and the majority have provided biospecimens for Project 2. Additional engagement and recruitment strategies are being deployed and evaluated for efficacy to promote increased enrollment of diverse participants.

**Conclusion:**

The multidisciplinary study of 80+ year‐olds with exceptional memory including imaging, blood draw, and unsupervised remote data collection using wearable technologies is feasible. The prospective study of SuperAgers is uncommon but holds promise for identifying mechanisms of resilience and resistance. Outcomes have potential relevance for isolating modifiable factors for promoting healthspan and avoiding ADRD.

